# Safety, Tolerability, and EEG-Based Target Engagement of STP1 (PDE3,4 Inhibitor and NKCC1 Antagonist) in a Randomized Clinical Trial in a Subgroup of Patients with ASD

**DOI:** 10.3390/biomedicines12071430

**Published:** 2024-06-27

**Authors:** Craig A. Erickson, Laura Perez-Cano, Ernest V. Pedapati, Eric Painbeni, Gregory Bonfils, Lauren M. Schmitt, Hannah Sachs, Meredith Nelson, Lisa De Stefano, Grace Westerkamp, Adriano L. S. de Souza, Oliver Pohl, Offir Laufer, Gil Issachar, Thomas Blaettler, Jean-Marc Hyvelin, Lynn A. Durham

**Affiliations:** 1 Division of Child and Adolescent Psychiatry, Cincinnati Children’s Hospital Medical Center, Cincinnati, OH 45229, USA; 2 Department of Psychiatry and Behavioral Neuroscience, University of Cincinnati, Cincinnati, OH 45229, USA; 3 Discovery and Data Science (DDS) Unit, STALICLA SL, Moll de Barcelona, s/n, Edif Este, 08039 Barcelona, Spain; 4 Division of Child Neurology, Cincinnati Children’s Hospital Medical Center, Cincinnati, OH 45229, USA; 5 Drug Development Unit (DDU), STALICLA SA, Campus Biotech Innovation Park, Avenue de Sécheron 15, 1202 Geneva, Switzerland; 6 Division of Behavioral Medicine and Clinical Psychology, Cincinnati Children’s Hospital Medical Center, Cincinnati, OH 45229, USA; 7 Department of Pediatrics, College of Medicine, University of Cincinnati, Cincinnati, OH 45229, USA; 8 Firefly Neuroscience, Herzliya 4672501, Israel

**Keywords:** ASD-Phen1, STP1, ibudilast, bumetanide, phase 1b, EGG, NIH-TCB, SRS-2, ABC-C

## Abstract

This study aimed to evaluate the safety and tolerability of STP1, a combination of ibudilast and bumetanide, tailored for the treatment of a clinically and biologically defined subgroup of patients with Autism Spectrum Disorder (ASD), namely ASD Phenotype 1 (ASD-Phen1). We conducted a randomized, double-blind, placebo-controlled, parallel-group phase 1b study with two 14-day treatment phases (registered at clinicaltrials.gov as NCT04644003). Nine ASD-Phen1 patients were administered STP1, while three received a placebo. We assessed safety and tolerability, along with electrophysiological markers, such as EEG, Auditory Habituation, and Auditory Chirp Synchronization, to better understand STP1’s mechanism of action. Additionally, we used several clinical scales to measure treatment outcomes. The results showed that STP1 was well-tolerated, with electrophysiological markers indicating a significant and dose-related reduction of gamma power in the whole brain and in brain areas associated with executive function and memory. Treatment with STP1 also increased alpha 2 power in frontal and occipital regions and improved habituation and neural synchronization to auditory chirps. Although numerical improvements were observed in several clinical scales, they did not reach statistical significance. Overall, this study suggests that STP1 is well-tolerated in ASD-Phen1 patients and shows indirect target engagement in ASD brain regions of interest.

## 1. Introduction

Autism Spectrum Disorder (ASD) comprises a group of lifelong neurodevelopmental conditions that, according to recent estimates, affect 1.5% of the population in developed countries [1]. In 2020, the Autism and Developmental Disabilities Monitoring Network reported that the prevalence of ASD in the United States was 3.8-fold higher among boys as compared to girls (43.0 versus 11.4) [2]. According to the current diagnostic criteria from the DSM-5 (Diagnostic and Statistical Manual of Mental Disorders, 5th Edition) [3], an individual with ASD must show deficits in social interaction and communication combined with at least two of four subdomains of restricted or repetitive behaviors. The manifestations of ASD must cause clinically significant impairment, affecting the ability of patients to interact with others, especially same-aged peers in youth.

The current version of the DSM-5 abandoned classifications of subtypes of ASD to group them under one umbrella, mostly due to the lack of objective criteria to define previous subtypes, including Asperger’s Disorder and Pervasive Developmental Disorder Not Otherwise Specified (PDD-NOS) [4]. However, ASD remains characterized by high heterogeneity in its behavioral manifestations, with very complex genetic underpinnings, suggesting the existence of subtypes of ASD [5]. Therefore, efforts to categorize ASD are still of critical importance and must rely on defining a relationship between clinical symptoms and biological mechanisms to tailor treatments to patient categories and thereby improve treatment benefit in these patients. Along these lines, STALICLA’s Databased Endophenotyping Patient Identification (DEPI^®^) technology was developed as a systems biology-based, artificial intelligence (AI)-driven first-in-class platform enabling matching biologically defined populations of patients with neurodevelopmental disorders (NDDs) with tailored treatments, with a first application to ASD [6].

Application of DEPI led to the identification of a first clinically and biologically defined subgroup of patients with ASD, ASD Phenotype 1 (ASD-Phen1) [7]. The ASD-Phen1 subgroup was predicted to be characterized by the presence of specific, non-behavioral, clinical signs and symptoms (CSSs) (i.e., an enlarged head circumference during early infancy combined with aggravation of core ASD symptoms during episodes of immune activation), mirroring the effects of a convergent molecular pathophysiology linked to an over-activation of Nuclear factor kappa-light-chain-enhancer of activated B cells (NF-κB) and Nuclear factor erythroid 2-related factor 2 (NRF2) transcription factors and their related pathways. By conducting a novel observational and bio-sampling study, we were able to validate the existence of this subgroup of patients with an observed prevalence of ~24% in 84 patients who qualified to be enrolled in the study [7]. Blood samples collected from this study’s participants also confirmed the presence of metabolic and transcriptomic alterations differentiating patients with ASD-Phen1 that were consistent with an over-activation of NF-κB and NRF2 transcription factors as predicted by DEPI. Importantly, further pre-clinical work led to the identification of STP1, a combination of ibudilast (a brain penetrant phosphodiesterase 3 (PDE3), 4 (PDE4), 10 (PDE10), and 11 (PDE11) inhibitor [8,9]) and bumetanide (an inhibitor of the sodium-potassium-chloride co-transporter 1 and 2 (NKCC1/NKCC2) [10]), as a potential tailored treatment with capacity to revert such observed molecular alterations in patients with ASD-Phen1 [7]. At the cellular level, treatment with ibudilast induces an increase in cyclic adenosine monophosphate (cAMP) and cyclic guanosine monophosphate (cGMP), leading to activation of intracellular signaling cascades (e.g., protein kinase A [PKA]) and resulting in increased expression of the Na–K–Cl cotransporter 1 (NKCC1). At the physiological level, these increased expression levels and activation of NKCC1 could lead to accumulation of intracellular chloride in cells and antagonize the gamma-aminobutyric acid (GABA) current in neurons, thereby decreasing inhibition and favoring excitation. This potential increased neuronal excitation effect is expected to be compensated by bumetanide [8,9,10,11,12].

Building on the insights gained from DEPI analysis of the observational research study, we conducted a phase 1b study in twelve patients matching the clinical criteria for ASD-Phen1. The main objective of this study was to confirm the safety and tolerability of STP1. In addition, the study aimed at exploring the clinical and electrophysiological effects of STP1 in the ASD-Phen1 patient population to validate our precision medicine-based approach in ASD.

## 2. Materials and Methods

### 2.1. Diagnosis and Main Criteria for Eligibility

Subjects matching the ASD-Phen1 definition [7] were pre-identified as part of a separate observational biosampling study (NCT04273087) conducted in the same clinical center.

Patients were eligible to participate in the study if they met all of the following inclusion criteria: (1) male or female individuals, aged between 18 and 40 years inclusive, previously diagnosed with ASD (based on a medical history of matching DSM-5 criteria and confirmed by an interview with an expert in autism diagnosis (C.A.E.)); (2) a well-documented enlarged head size at any time point after birth and before 24 months of age, defined as a head circumference (HC) ≥ 75th percentile according to the Centre for Disease Control and Prevention (CDC) growth charts, or a diagnosis of macrocephaly; (3) a systematic aggravation of ASD core behavioral symptoms, so-called flares, occurring during episodes of immune challenges (such as fever and infectious events like acute inflammation), assessed by the ASD-Phen1 semi-structured interview form developed by the sponsor according to the DEPI^®^ platform-derived criteria; (4) a parent or reliable caregiver who agrees to provide information about the patient as required by the protocol; (5) the patient willing and consenting or assenting to participate as applicable, and if assent provided by the patient, parent or legal guardian also willing to give written consent; (6) patients with ASD and comorbid seizure disorder should be seizure-free for at least six months prior to screening; (7) before entering the study, patients have to agree to use double-barrier birth control methods if they are to engage in intercourse; (8) women of childbearing potential, defined as all women physiologically capable of becoming pregnant, could be enrolled if they were using highly effective methods of contraception during dosing and for one week after discontinuation of the investigational drug.

Patients were excluded if they met any of the following exclusion criteria: (1) patients with an identified genetic cause of ASD in their medical record; (2) history of traumatic head injury, cerebrovascular disorder, congestive heart failure, hepatic, diabetes, or renal disease or thrombocytopenia (1 year before the screening visit) or significant abnormal laboratory tests.

Additional exclusion criteria included use of prohibited medications, antioxidant supplements, vitamins, or herbal remedies within two weeks prior to randomization, or five half-lives (whichever was longer); alcohol and/or substance abuse/dependence within 12 months prior to screening; any active infection, suicidal risk, or any history of malignancy of any organ system; or any episode of fever or illness without fever within 10 days before the treatment.

During the study, the inclusion criteria were adapted to satisfy regulatory requirements. In addition, diagnosis of macrocephaly was added to inclusion criterion (2), as part of a protocol amendment, to enable the identification of ASD-Phen1 patients with no available information on head circumference during the first 2 years of life, provided that an enlarged head circumference in infancy was expected to persist throughout adulthood for these patients [13].

### 2.2. Drug Manufacturing

STP1 refers to the combination of ibudilast and bumetanide. A combination of the two drugs was concomitantly administered orally in this study in patients identified as ASD-Phen1. Ibudilast sustained-release capsules were manufactured by Emerson Resources, Inc. (Norristown, PA, USA) in two strengths, 5 mg (batch #P3954-23) or 10 mg (batch #P3954-34). Bumetanide is available as tablets, in dosage strengths of 0.5, 1, and 2 mg for b.i.d. administration. Commercially available bumetanide tablets (1 mg) were acquired from Amneal Pharmaceutical (Bridgewater, NJ, USA).

### 2.3. Study Design and Dosing

This was a single-center randomized, double-blind, placebo-controlled, parallel-group, 2-dose ascending study. Patients were randomized 3:1 to either STP1 or matching placebo b.i.d. treatment for 14 days (a.k.a. Cohort 1). The 2-week treatment period was followed by a 2-week follow-up period. Dose escalation and enrollment of Cohort 2 was conducted following a Data and Safety Monitoring Board (DSMB) review of Cohort 1 safety and tolerability data. The short 2-week treatment duration was chosen considering that this was the first-in-man clinical study of STP1 while still maintaining the potential of observing a short-term pharmacodynamic effect.

The screening visit included written informed consent, updated medical history, physical examination, drugs of abuse screening, pregnancy test (females only), alcohol test, laboratory data, Electrocardiogram (ECG) safety, coagulation, viral serology, urinalysis, and inclusion/exclusion criteria. Patients were to return to the study site at scheduled visits on Day 1, Day 7, Day 14/15, and optionally up to Day 18 for evaluation and PK sampling, and at approximately 14 days (Day 28) after the last dose for a post-treatment safety follow-up visit.

For each dose cohort, the total duration of the study for each participant was up to six weeks, divided as follows: a screening phase of up to two weeks (from Day −14 to Day −1); a double-blind treatment phase of two weeks (from Day 1 to Day 14); and a follow-up phase of two weeks after treatment discontinuation (from Day 15 to Day 28).

The study was blinded for patients, caregivers, and the study personnel with direct interaction with the subjects, using placebo capsules and tablets that matched the active treatments in appearance, labeling, and packaging. The total number of capsules and tablets was identical across STP1 dose levels and placebo.

In Cohort 1, patients were to receive SPT1 5/1 mg per os b.i.d. (i.e., 5 mg ibudilast and 1 mg bumetanide, *n* = 6) or a matching oral placebo (*n* = 2). Once the first dose cohort (eight patients) finished the two weeks of STP1 treatment and the two weeks of follow-up, a DSMB was to evaluate the safety data before allowing the enrollment of patients in Cohort 2. Cohort 2 was to receive STP1 10/1 mg per os b.i.d. (i.e., 10 mg ibudilast and 1 mg bumetanide, *n* = 3), or a matching oral placebo (*n* = 1).

The capsules and tablets were to be taken at the same time with food and water, and the two daily doses were separated by approximately six hours. For all patients, twice daily dosing was to occur on Days 1 to 13, inclusive, and a final single dose administration occurred on the morning of Day 14 (morning dose only). On Days 1, 7, and 14, the morning dose was to be taken on site.

The starting dose of 5 mg of ibudilast twice a day (6 h apart) was chosen based on the PK and safety data available from the literature and the clinic [7]. The margin of safety (MOS) associated with the starting dose is 5 and was derived from the preclinical toxicokinetic data and the estimated NOAEL in the toxicology studies performed in dogs, the most sensitive species to ibudilast in those studies. The second dose level of 10 mg twice a day (6 h apart) was expected to allow ibudilast to reach a Ctrough of approximately 13 ng/mL, corresponding to 56 nmol, which is within the range of PDE4 IC50 [9]. Hence, the 5 and 10 mg twice a day (6 h apart) doses were expected to have modulatory activity on PDE4 and to possibly show positive electrophysiological and/or clinical PD effects.

Bumetanide alone given to children and adolescents with ASD, aged 2 to 18 years, has been shown to improve core symptoms in a subset of patients, with an acceptable risk/benefit ratio at a dose of 1 mg twice a day (6 h apart) for 3 months [14,15].

### 2.4. Primary Outcomes

Primary endpoints aimed to evaluate the safety and tolerability of STP1 and included (i) the incidence, nature, and severity of adverse events (AEs) and severity of serious adverse events (SAEs); (ii) the change from baseline in DBP (diastolic blood pressure), SBP (systolic blood pressure), heart rate (HR), and respiratory rate; and (iii) the incidence of clinically relevant laboratory abnormalities, based on hematology, coagulation, blood chemistry, ECG, and urinalysis test results.

### 2.5. Secondary Pharmacokinetics (PK) Outcomes

Blood was sampled for the determination of plasma concentration of ibudilast and bumetanide, and urine samples were taken. For placebo subjects, only Day 1 (1 h and 6 h), Day 7 (pre-dose), and Day 14/15 (1 h and 6 h to determine PK characteristics) samples were analyzed.

### 2.6. Exploratory Pharmacodynamic (PD) Outcomes

- Electrophysiological Parameters

For Electroencephalogram (EEG) acquisition and preprocessing an Magstim EGI NetAmps 400 amplifier system was used to acquire EEG data using128-channel HydroCel saline-based electrode nets (Eugene, OR, USA) at a sampling rate of 1000 Hz. EEG data were blinded and semi-autonomously preprocessed. After visualization of raw data, bad channels were interpolated using spherical spline interpolation. EEG data were first downsampled to 500 Hz and average referenced. High- and low-pass cutoff frequencies were set at 2 and 120 Hz and a 60 Hz notch filter was used for the removal of power-line noise. Physiological artifacts (eye movement, cardiac, and muscle movement) were removed using independent component analysis (ICA; extended infomax) in EEGLAB [16] using MATLAB 2021A (MathWorks, Natick, MA, USA).

Resting EEG, i.e., the change from baseline in resting-EEG gamma power (dB, absolute and relative) in several brain areas associated with cognition and emotional processing (frontal poles, entorhinal, superior temporal), was calculated by subtracting the group-averaged pre-dose scores. The changes in resting-EEG power were compared between cohorts.

Eighty seconds of continuous artifact-free data were included from each session for analysis following preprocessing. This artifact-free continuous data were segmented into 2 s epochs, detrended, and tapered with a Hanning window. Fast Fourier transform was used to generate Fourier coefficients in 0.5 Hz frequency steps and squared to yield power values (μV2). Frequencies were divided into six frequency bands: delta (2–3 Hz), theta (4–7 Hz), lower alpha (8–10 Hz), upper alpha (10–12 Hz), beta (13–30 Hz), and gamma (30–80 Hz). To calculate relative power, the relative power of each frequency band was computed as the fraction of the sum of power measurements from 2 to 80 Hz [17].

For auditory chirp (a.k.a. auditory synchronization) the current experiment utilized a chirp stimulus to evaluate neural synchronization to sensory input across a range of frequencies from 1 to 100 Hz (gamma waves). Participants heard an auditory chirp stimulus generated using a 1000 Hz tone that was amplitude-modulated by a sinusoid linearly increasing in frequency from 1 to 100 Hz over 2 s. Data were epoched into 3250 ms trials (−500 to 2750 ms). Trials with a post-ICA (Independent Component Analysis) amplitude exceeding 120 μV were rejected. Data from 23 sensors distributed across the fronto-central scalp were used as an a priori region of interest to capture auditory cortex signals [18]. Single-trial power (STP) and inter-trial coherence (ITC) were calculated using Morlet wavelets with a 1 Hz frequency step using a linearly increasing cycle length from 1 cycle at the lowest frequency (2 Hz) to 30 cycles at the highest (120 Hz). ITC was calculated after baseline correction using the 500 ms pre-stimulus period. Raw ITC values were also corrected by the trial number by subtracting the critical r value as calculated by sqrt [−1/(number of trials) × log(0.5)]. Final STP and ITC values were averaged over trials for each individual and transformed into time–frequency plots.

b. Clinical parameters

For the PD clinical parameters, change from baseline to Day 14 was measured for the following assessments: sub-tasks from the National Institutes of Health Toolbox Cognition Battery (NIH-TCB) were used to assess cognition, in particular the crystalized composite that included two subtests, picture vocabulary and oral reading, to assess cognition dependent upon past experiences; Aberrant Behavior Checklist-Community (ABC-C) sub-scores were used to assess behavioral disturbances; Social Responsiveness Scale 2nd Edition (SRS-2) sub-scores to assess social abilities; and Clinical Global Impression-Severity (CGI-S) scale, reflected by the Clinical Global Impression-Improvement (CGI-I) scale, to assess symptom severity and treatment response.

### 2.7. Analysis Populations

The primary objective of this study was to evaluate the safety and tolerability profile of 2 dose levels of STP1 in patients with ASD-Phen1. Up to six patients as active and up to 2 placebos per dose level (maximum 4 on placebo total) were considered sufficient for the primary objective.

The safety analysis, pharmacokinetic analysis, and exploratory PD analysis populations were defined as follows. The safety analysis population consisted of all patients who had received at least one administration of the study treatments, whether prematurely withdrawn from the study or not. For the safety analysis population, data were analyzed according to the treatment taken.

The pharmacokinetic (PK) analysis population consisted of all patients with at least one administration of the study treatments and with at least one sample collected and analyzed for plasma drug concentration, whether prematurely withdrawn from the study or not.

The exploratory PD analysis population consisted of all randomized patients who met the inclusion/exclusion criteria, received a full course of the study drug as per the randomization scheme, had completed the main relevant visits, and had no major protocol violations, which would render the data unreliable.

For this phase 1b study, descriptive statistics were provided for all variables by treatment group (placebo data were pooled from the two dose cohorts). For continuous endpoints, summary statistics, i.e., *n* (number of non-missing observations), mean, standard deviation (SD), median, minimum, and maximum were provided. For categorical variables, the frequency count and proportion in each category were summarized.

Some assessment time points varied in line with a protocol amendment that was in place at the time of enrollment. Data from all time points were summarized as appropriate. All data collected were presented in the by-patient data listing, sorted by patient and by time point where appropriate. No missing value imputation was used. That is, all analyses were based on the observed data (i.e., a complete case analysis).

Change from baseline in resting-EEG gamma power was calculated and compared across cohorts, using a 1-way Analysis of Variance (ANOVA) test, followed by Tukey pairwise post hoc tests. In addition, EEG topographic maps were plotted, providing whole-brain visualization of changes from baseline in gamma power. Pearson correlation analyses were conducted between resting-EEG gamma power in regions of interest and STP1 concentrations pooled across Day 1 and Day 14.

Changes from baseline across treatment periods of auditory chirp response variables (gamma STP, ITC 40 Hz, ITC 80 Hz, and ITC onset) were compared using a 2-way repeated measures ANOVA.

## 3. Results

### 3.1. Patient Disposition and Demographics

**Figure 1:**
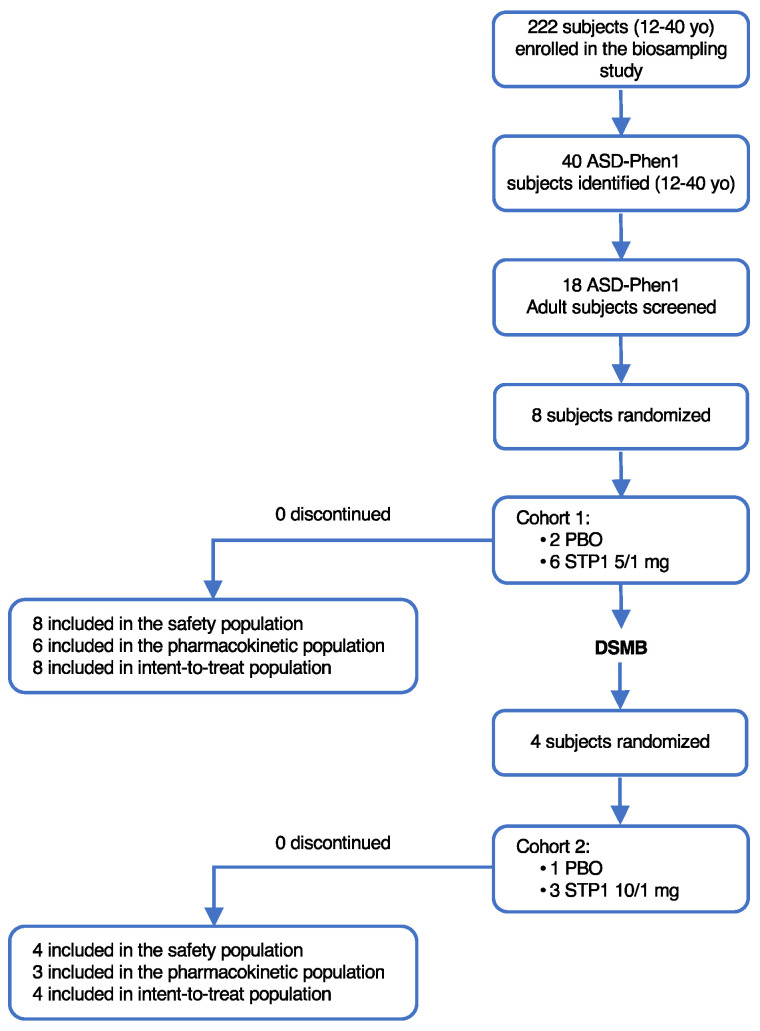
Patient disposition.

The ASD-Phen1 patients were identified from a previous biomarker study (ClinicalTrials.gov Identifier: NCT04273087). Among the 222 subjects with idiopathic ASD enrolled in the biomarker study, 40 were identified as matching the clinical criteria for ASD-Phen1, resulting in an observed ASD-Phen1 prevalence of ~18% within this cohort, in line with the prevalence observed in a previous study [6]. Among these 40 subjects, 18 adults were invited for participation in the interventional phase 1b study (Figure 1). Six male and two female patients 18 years of age or older were included in Cohort 1 and randomly assigned in a 3:1 ratio to STP1 low dose or placebo through randomized treatment assignment using SAS PROC PLAN software (SAS, V9.4). The enrollment occurred at a single US site, the Cincinnati Children’s Hospital Medical Center. Following the review of the Cohort 1 data, the Data and Safety Monitoring Board (DSMB) recommended the initiation of Cohort 2. Four patients (3 males, 1 female) from Cohort 1 were re-randomized in a 3:1 ratio to high-dose STP1 or placebo in Cohort 2. For those four patients, the shortest period between the last dose in Cohort 1 and the start of dosing in Cohort 2 was eight weeks. In both cohorts, all patients completed the study, including the 2-week treatment period and the 2-week follow-up period (Figure 1).

Overall, a total of seven male and two female patients received STP1 treatment (75% of patients). Patient demographics are summarized in Table 1. Of note, patients participating in Cohort 1 and 2 are counted twice.

The mean age, height, and head circumference (within the first two years of age) were similar in all groups. The mean weight and Body Mass Index (BMI) were lower in the placebo group than in the STP1 5/1 mg and STP1 10/1 mg groups. The mean weight (kg) was 114.22 kg in the STP1 5/1 mg group, 109.80 kg in the STP1 10/1 mg group, and of 78.13 kg in the placebo group. The mean BMI was 36.80 in the STP1 5/1 mg group, 37.07 in the STP1 10/1 mg group, and 25.17 in the placebo group.

### 3.2. Adverse Events

STP1 was generally well tolerated. All adverse events were assessed as being mild and occurred with a similar incidence across treatment groups (Table 2). A total of four subjects (67%) experienced at least one adverse event in the STP1 5/1 mg group, with one subject (17%) having an adverse event that was treatment related (pollakiuria). Two subjects (67%) in the STP1 10/1 mg experienced at least one adverse event. There were two subjects (67%) in the placebo group that experienced at least one adverse event.

There were no reported serious adverse events (SAE) and no reported adverse events of special interest (AESI) in the study overall. There were no deaths reported in the study overall.

### 3.3. Pharmacokinetic Analysis

- PK profile of STP1 in Blood

**Figure 2:**
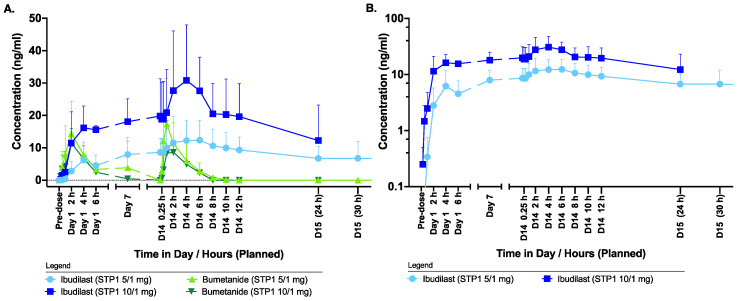
Pharmacokinetic profiles of ibudilast and bumetanide in ASD-Phen1 dosed with STP1 5/1 mg (i.e., 5 mg ibudilast plus 1 mg bumetanide, *n* = 6) or STP1 10/1 mg (i.e., 10 mg ibudilast plus 1 mg bumetanide, *n* = 3), twice a day during a 2-week treatment period. (**A**) shows concentrations of ibudilast and bumetanide using linear scale; (**B**) shows concentration of ibudilast using log scale. Note: On Day 14, patients received morning dose only. Blood samples were collected for PK assessment on Day 1 (pre-dose and 0.25, 0.5, 1, 2, 4, 6 h, post-dose), on Day 7 (pre-dose), on Day 14 (pre-dose and 0.25, 0.5, 1, 2, 4, 6 h, post-dose), and on Day 15 (0 h and 6 h, corresponding to 24 h and 30 h after the last administered dose on Day 14). Undetectable concentrations of ibudilast and bumetanide are depicted as half of the lower limit of quantitation (LLOQ) for the analytical method applied for each drug, namely ½ of 0.200 = 0.100 ng/mL for ibudilast and ½ of 2 = 1.00 ng/mL for ibudilast for the calculation of the means. When the means were below the LLOQ, a null value was plotted. Symbols indicate mean; bars indicate standard deviation (SD).

Ibudilast and bumetanide mean plasma concentration levels by treatment are shown in Figure 2. The sampling time points were selected to allow for the assessment of the PK profile after the first dose on Day 1 and at the expected steady state on Day 7 and Day 14. In addition, the elimination of both drugs was characterized up to a 30 h sampling period after the last dose on Day 14 (Figure 2).

Maximum observed ibudilast concentrations were reached 4 h after administration for the two STP1 doses both at Day 1 and at Day 14.

Compared to the STP1 5/1 mg group, ibudilast plasma concentrations in the STP1 10/1 mg group were approximately two-fold increased at the corresponding time points.

Bumetanide reached plasma T_max_ within 1–2 h after dose administration. The bumetanide plasma concentrations for the STP1 5/1 mg and STP1 10/1 mg group were similar, as the same dose of bumetanide was administered, in line with no noticeable PK interaction between the two drugs. Whereas bumetanide was completely eliminated within 10 h of the last administration, low plasma levels of ibudilast were still present in eight out of nine and in three out of three subjects at 24 and 30 h, respectively, after the last dose.

The summary statistics of estimated PK plasma parameters for ibudilast by treatment are provided in Table 3. Following STP1 5/1 mg and 10/1 mg administration, ibudilast had a high volume of distribution, indicating that the drug distributed well into body tissues. Both ibudilast 5 mg and 10 mg appeared to be rapidly cleared. The mean t_1/2_ of ibudilast 5 mg was 67.28 h, and the mean t_1/2_ of ibudilast 10 mg was 16.71 h on Day 14. The t_1/2_ observed for the high-dose group is in line with the value reported in the literature (19.2 h) [19].

Summary statistics of estimated PK plasma parameters for bumetanide by treatment are provided in Table 3. Overall, similar PK parameters were observed for bumetanide after STP1 5/1 mg or 10/1 mg administration. This similarity was in line with no effect of ibudilast on the PK parameters of bumetanide. Overall, bumetanide had a short half-life of about 2 h, with a moderate volume of distribution and rapid clearance. The calculated T_max_ was 1.76–2.07 h and 1.54–1.65 h on Day 1 and Day 14, respectively, in both STP1 5/1 mg and STP1 10/1 mg groups.

Despite this variability, the geometric mean AUC for ibudilast was comparable with published reports [19] for Cohort 2.

b. Excretion of ibudilast and bumetanide in urine following treatment with STP1

Overall, following SPT1 administration, unmetabolized ibudilast was absent in urine (i.e., <0.1% of the administered dose recovered over a 12 h period at Day 14).

The mean cumulative urine excretion for bumetanide in its unchanged form over a 12 h period at Day 14 was 334.01 mcg and 350.31 mcg for the STP1 5/1 mg and STP1 10/1 mg groups, respectively. These values correspond to 33–35% of the administered oral dose and are similar to the observation from the literature that 51.3% of a bumetanide dose is excreted unchanged in urine [16], possibly due to a shorter urine collection time of the present study (12 h vs. 48 h).

### 3.4. Exploratory Outcomes: PD Parameters

- NIH-TCB Crystalized Composite

**Figure 3:**
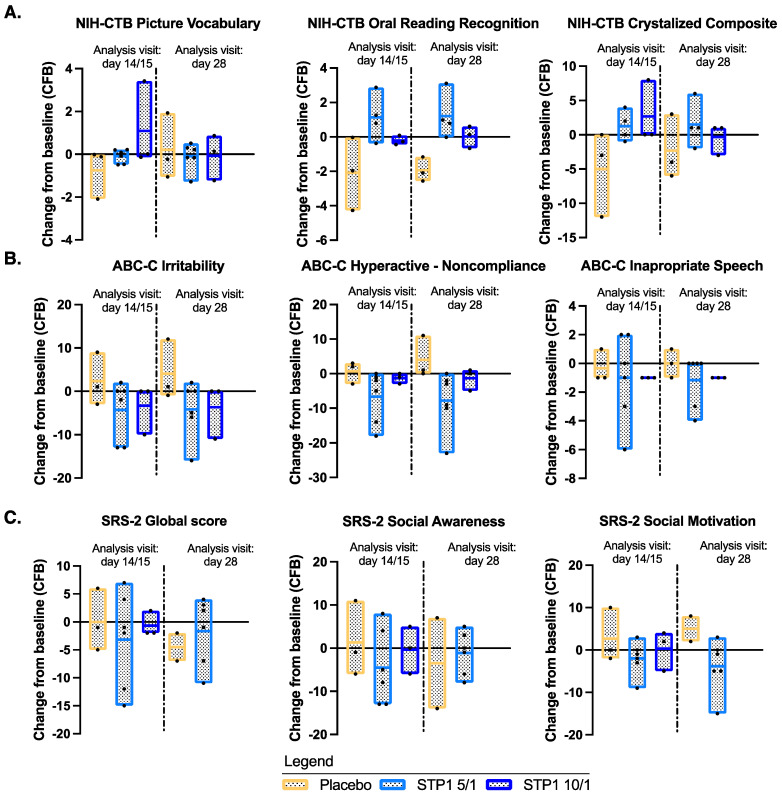
NIH-TCB, ABC-C, and SRS-2. (**A**) NIH-TCB-Picture Vocabulary, NIH-TCB-Oral Reading Recognition, and NIH-TCB-Crystalized composite uncorrected scores after administration of STP1 5/1 mg (*n* = 4–6), STP1 10/1 mg (*n* = 3), or placebo (*n* = 3), twice a day, on Day 14/15 and on Day 28 (i.e., 2 weeks following last dosage). Note: NIH-TCB oral reading recognition was acquired only in *n* = 4 patients treated with STP1 5/1; therefore, NIH-TCB crystalized composite was only computed in *n* = 4 patients treated with STP1 5/1; (**B**) Aberrant Behavior Checklist-Community (ABC-C) sub-scales in ASD-Phen1 patients dosed with STP1 5/1 mg (*n* = 6), STP1 10/1 mg (*n* = 3), or placebo (*n* = 3) on Day 14/15 and Day 28. Note: On Day 28, SRS-2 sub-scales were not collected for the Cohort 2 with STP1 10/1 mg; (**C**) Social Responsive sub-scales in ASD-Phen1 patients dosed with STP1 5/1 mg (*n* = 6), STP1 10/1 mg (*n* = 3), or placebo (*n* = 3) on Day 14/15 and Day 28. Data are expressed as change from baseline (CFB) (i.e., score measured during screening visit). Column bar represents min–max and mean, and dots represent individual values. Additional plots for NIH-CTB, ABC-C, and SRS-2 are included in Supplementary Figure S1.

NIH-TCB Crystalized composite (NIH-TCB-picture vocabulary and NIH-TCB-oral reading) showed a numerical increase (indicative of improvement) from baseline for the Crystalized Composite Fully Uncorrected Standard Score for subjects in the STP1 5/1 mg and in the STP1 10/1 mg groups relative to baseline on Day 14/15 (Figure 3A), whereas a decrease was observed in the placebo group. This apparent effect was no longer observable on Day 28, i.e., 2 weeks after the end of the treatment. The NIH-TCB-picture vocabulary showed a minimal increase from baseline for subjects in the STP1 5/1 mg and in the STP1 10/1 mg groups on Day 14/15 (Figure 3A); NIH-TCB-Oral Reading Recognition showed a numerical increase (indicative of improvement) from baseline for subjects in the STP1 5/1 mg group on Day 14/15 (Figure 3A). None of the above-described observations reached statistical significance.

b. Exploratory Outcomes: Aberrant Behavior Checklist-Community (ABC-C) and Social Responsiveness Scale, 2nd Edition (SRS-2)

For ABC-C, there was a numerical decrease (indicative of improvement) in the mean change from baseline score at Day 14/15 in the STP1 5/1 mg and STP1 10/1 mg groups compared to the placebo and maintained at day 28. The most noticeable directional improvements in the STP1 groups appeared to be in Irritability, Hyperactivity, and Inappropriate Speech sub-scales (Figure 3B).

As for the SRS-2 global score, the STP1 5/1 mg group showed a numerical decrease (indicative of improvement) in the mean change from baseline score on Day 14/15, whereas the placebo group and the STP1 10/1 mg group showed no change. This effect in the low-dose group was primarily seen in social awareness, social motivation, and mannerisms (Figure 3C). No statistical significance was reached.

c. Exploratory Outcomes: EEG-Gamma Bands

**Figure 4:**
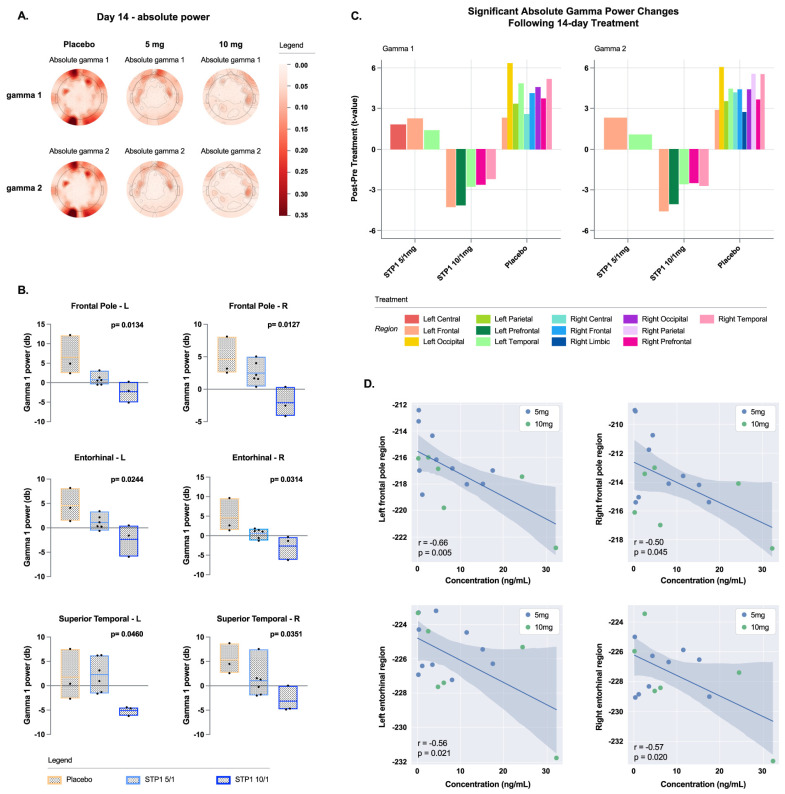
Gamma bands in ASD-Phen1 patients dosed with STP1 5/1 mg (*n* = 6) (i.e., ibudilast 5 mg plus bumetanide 1 mg), STP1 10/1 mg (*n* = 3) (i.e., ibudilast 10 mg plus bumetanide 1 mg) or placebo (*n* = 3), twice a day, on Day 14 (end of treatment). (**A**) Topoplots showing change in absolute power in gamma 1 (**top**) and gamma 2 (**bottom**); (**B**) gamma 1 power (dB) in Frontal and Entorhinal brain regions at Day 14 (each value is normalized to pre-dose); (**C**). Scalp EEG gamma changes in placebo, STP1 5/1 mg, and STP1 10/1 mg. Only participants with both pre and post treatment within hemispheric region were considered, *n* = 2 placebo, *n* = 5 STP1 5/1 mg, and *n* = 3 STP1 10/1 mg. Values less than 0 indicate a drop in gamma power following treatment. For this analysis only, participants with pre and post timepoints were considered (one patient in placebo group and one patient in STP1 5/1 mg were excluded); (**D**) correlation between EEG gamma power (dB) and ibudilast C_max_ concentration (ng/mL) measured at Day 1 and at Day 14 post-dose in cohorts STP1 5/1 mg (*n* = 10) and STP1 10/1 mg (n = 6). Pearson’s r and *p* values are presented for each correlation. Shaded areas indicate 95% CI.

Resting EEG shows a dose-related effect of STP1 in reducing gamma power (dB, absolute and relative, Figure 4A).

d. Exploratory Outcomes; Gamma Bands in Brain Regions of Interest

In brain regions of interest associated with executive function (frontal left and right poles) and memory (left and right entorhinal regions), STP1 induced a statistically significant dose-related effect in reducing gamma power in comparison to the placebo (Figure 4B). Specifically, a significant effect was observed in brain regions of interest associated with social cognition and emotional processing for the left and right frontal poles (executive function; F(2,9) = 7.23, *p* = 0.013, eta-squared = 0.616 and F(2,9) = 7.37, *p* = 0.012, eta-squared = 0.621, respectively), left and right entorhinal regions (memory-related processes; F(2,9) = 5.76, *p* = 0.024, eta-squared = 0.561 and F(2,9) = 5.21, *p* = 0.031, eta-squared = 0.536, respectively), and right superior temporal region (indirect target engagement; F(2,9) = 4.97, *p* = 0.035, eta-squared = 0.525).

The resting state data were reanalyzed using source estimation to confirm scalp EEG gamma changes with treatment. Previously published methods [20] were used to generate a minimal norm estimate (MNE) electrical model of EEG activity. For this analysis, only participants with both a pre- and 14-day post-timepoint were included. The bar plot shows that STP 10/1 mg is associated with a drop in gamma power across multiple cortical regions (Figure 4C).

Correlation analyses between resting-EEG gamma power in the regions of interest and ibudilast concentrations pooled across Day 1 and Day 14 revealed significant negative correlations in left and right frontal poles (r = −0.66, *p* = 0.005, and r = −0.50, *p* = 0.045, respectively), left and right entorhinal regions (r = −0.56, *p* = 0.021, and r = −0.57, *p* = 0.020, respectively), and right superior temporal region (r = −0.58, *p* = 0.017) (Figure 4D).

e. Exploratory Outcomes; EEG-Alpha Bands

**Figure 5:**
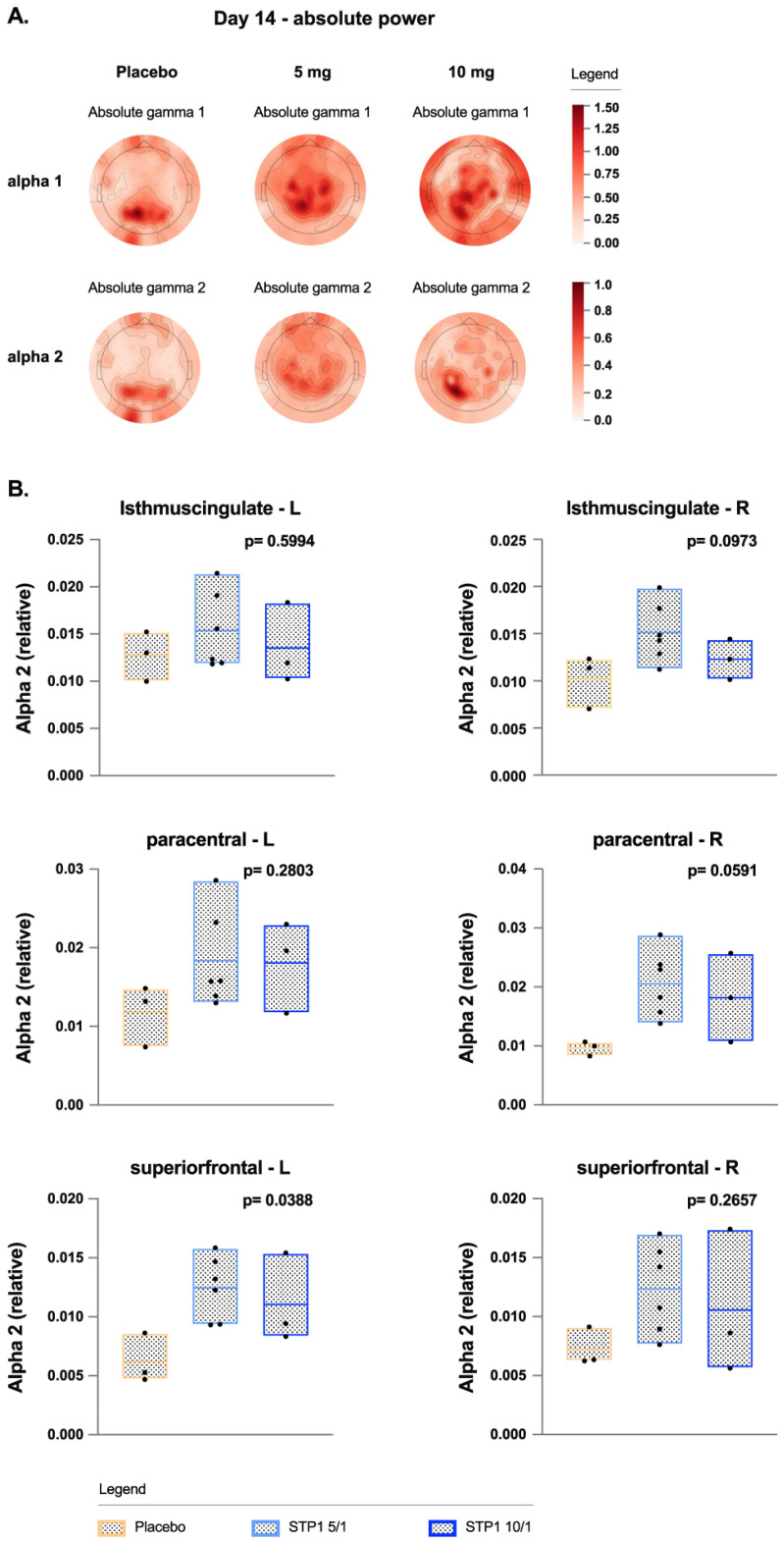
Alpha bands in ASD-Phen1 patients dosed with STP1 5/1 mg (*n* = 6) (i.e., ibudilast 5 mg plus bumetanide 1 mg), STP1 10/1 mg (*n* = 3) (*i.e.*, ibudilast 10 mg plus bumetanide 1 mg), or placebo (*n* = 3), twice a day, on Day 14 (end of treatment). (**A**) Topoplots showing change in absolute power in alpha 1 and alpha 2; (**B**) alpha 2 (relative) in brain regions on Day 14 (each value is normalized to pre-dose).

In ASD-Phen1, treatment with STP1 increased alpha waves (Figure 5) in the occipital and prefrontal regions, similar to other studies [21]. Treatment with STP1 showed a significant increase of alpha 2 power in frontal and occipital regions and in brain areas associated with memory function (frontal right poles, right isthmus cingulate) and sensory function (right paracentral regions).

f. Exploratory Outcomes: EEG-Complex Auditory Synchronization

**Figure 6:**
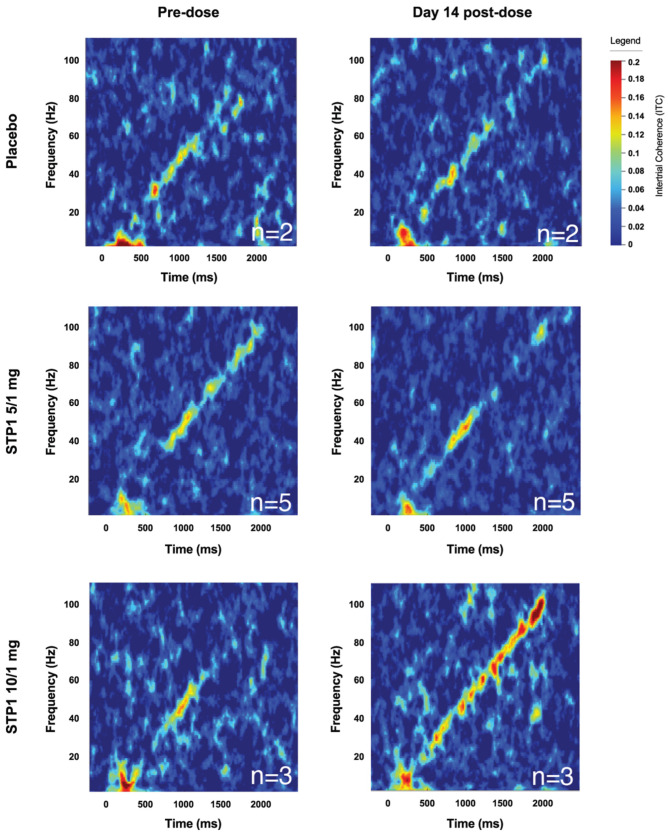
Complex synchronization analysis showing an increased ITC signal indicates increased neural synchronization to the auditory chirp measured in placebo *n* = 2, STP1 5/1 mg *n* = 5, and STP1 10/1 mg *n* = 3 at Day 1 post-dose and Day 14.

For the chirp analysis, 10 complete datasets were considered. Two datasets were incomplete due to missing baseline chirp EEG data. Responses include intertrial phase coherence (ITC; 40 Hz, 80 Hz, onset, and offset) and single trial power (STP; alpha, gamma 1, and gamma 2). Increased ITC indicates increased neural synchronization to the auditory chirp. STP refers to background spectral power during the auditory chirp. The ITC values are approximated from time–frequency analysis of the chirp response depicted in Figure 6. Overall, no clear numerical trends were identified in the STP1 5/1 mg group in regard to ITC.

In the STP1 10/1 mg group (*n* = 3), the provided mean score refers to change from baseline to Day 1 (acute post-dose), Day 14, and Day 28. Mean score for follow-up Day 14 includes ITC 40 Hz (0.008, SD = 0.086) and ITC 80 Hz (0.030, SD = 0.053); STP mean scores for follow-up Day 14 include alpha (−1.3, SD = 6.8), gamma 1 (−2.1, SD = 2.7), and gamma 2 (−2.6, SD = 1.7). Overall, there was a (non-statistical) numerical increase in ITC 80 Hz neural synchronization, which can be visualized in Figure 6.

g. Exploratory Outcomes: PK/PD Modeling Analysis

**Figure 7:**
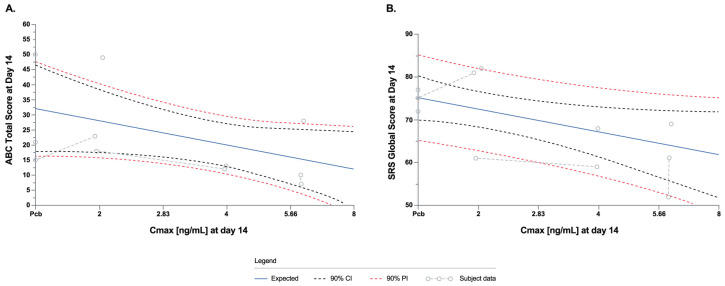
Linear PK/PD models using ABC-C scores (**A**) and SRS-2 scores (**B**) and ibudilast C_max_ (ng/mL) at Day 14.

PK/ PD modeling indicated a linear exposure/PD relationship for ABC-C and SRS-2. Based on the modeling data, a 5.3-point decrease (improvement) in the ABC-C total score would be predicted for a one log unit increase in C_max_ (Figure 7A). Similarly, an eight-point decrease (improvement) in the SRS-2 total would be predicted for a one log unit increase in C_max_ (Figure 7B).

## 4. Discussion

Screening of 222 patients with idiopathic ASD led to the identification of 40 patients matching the clinical criteria for ASD-Phen1, providing further clinical validation for the construct validity of ASD-Phen1, with a similar prevalence as previously observed [7]. Among those, eight adult patients with ASD-Phen1 agreed to participate in the interventional phase 1b study. Four out of the eight patients participated in Cohort 1 and Cohort 2. The main objective of this study was to assess the safety and tolerability of STP1. In this regard, STP1 treatment was well tolerated at the two dosages used. The tolerability of STP1 5/1 mg and STP1 10/1 mg was comparable with the placebo, with all AEs being assessed as being mild.

With regard to the PK characterizations, STP1 showed a similar PK profile for the individual components to the PK profiles described in the literature for ibudilast and bumetanide [19,22]. The high variability in PK parameters observed was driven by outliers that significantly impacted the means. As such, one of the limitations of the pharmacokinetics data was the low number of patients enrolled in the study. Therefore, in future studies, a larger number of participants and a wider dose range will be needed to fully characterize the PK of STP1 (ibudilast and bumetanide).

When it comes to the PD assessments, it is noteworthy that this trial was not powered to demonstrate a PD effect. Also, the short treatment duration of 2 weeks limited the ability to observe longer-term effects on functioning, generally expected to require longer treatment and observation periods. Nonetheless, we chose to explore a potential effect on the PD outcome measures. Among the PD outcome measures, EEG effects were explored as a surrogate for target engagement. Gamma oscillations hold a special interest in neurodevelopmental disorders because of their relation to cortical excitability [23,24], association with cognitive processes [25], and analogous measurability in animal models [26]. Importantly, resting EEG showed a significant dose-related effect of STP1 in reducing gamma power in brain regions of interest associated with working memory and flexible thinking processing for the left and right frontal poles (executive function), left and right entorhinal regions (related to memory), and right superior temporal region (understanding language, memory acquisition). Although not statistically significant, a numerical improvement for the chirp task (auditory synchronization) was observed, in which STP1 10/1 increased synchronization ability. Of note, although not formally assessed, the post-treatment evaluations on both electrophysiological assessments were comparable to electrophysiological findings in subjects without ASD. These effects in brain areas of interest may predict an improvement in sensory processing, which need to be validated in future studies with larger numbers of patients. Additionally, resting EEG showed significant increase of alpha power in brain regions of interest: Treatment with STP1 showed a significant increase of alpha 2 power in frontal and occipital regions and in brain areas associated with memory function (left superior frontal area, right isthmus cingulate) and sensory function (right paracentral regions). This aligned with the numerical cognitive effect observed on the NIH-crystalized composite. Previous reports have reported lower alpha waves in ASD. In a case report of a patient diagnosed with ASD, epilepsy, cortical dysplasia, and a 15q11.2 duplication, bumetanide treatment has been shown to increase alpha power and improve cognition [23]. Similarly, in patients diagnosed with tuberous sclerosis complex (which leads to multiorgan lesions, including neurodevelopmental disorders, e.g., intellectual disability, ASD-related behavior, epilepsy), bumetanide treatment has been shown to increase alpha power, which is correlated to an improvement in irritability [14].

In conclusion, this small exploratory study in individuals with ASD-Phen1 provides initial evidence of good tolerability of STP1. The electrophysiological effect on gamma and alpha 2 power in relevant brain regions and improvement of auditory synchronization are consistent with a pharmacodynamic effect on synaptic connectivity, which is further supported by directional improvements in the clinical exploratory endpoints. We hypothesize that these findings potentially predict a clinical benefit in learning and communication, which will need to be assessed in future larger studies with longer treatment duration and a broader dose range to fully characterize STP1 effects in the Phen1 subpopulation of ASD.

## Figures and Tables

**Table 1:** Demographics.

		STP1 5/1 mg (*n* = 6)	STP1 10/1 mg (*n* = 3)	Placebo (*n* = 3)	Overall ^a^ (*n* = 12)
Age (years), *n*		6	3	3	12
	Mean (SD)	18.67 (0.82)	19.67 (1.53)	19.67 (2.89)	19.17 (1.59)
Height (cm), *n*		6	3	3	12
	Mean (SD)	175.50 (10.60)	171.33 (14.15)	174.67 (11.15)	174.25 (10.64)
Weight (kg), *n*		6	3	3	12
	Mean (SD)	114.22 (23.46)	109.80 (31.23)	78.13 (26.32)	104.09 (28.32)
BMI (kg/m^2^), *n*		6	3	3	12
	Mean (SD)	36.80 (5.02)	37.07 (6.89)	25.17 (5.84)	33.96 (7.38)
HC 0–2 y (cm), *n*		5	2	3	10
	Mean (SD)	47.48 (5.03)	44.05 (7.85)	47.03 (4.27)	46.66 (4.91)
SB5-ABIQ, *n*		6	3	3	12
	Mean (SD)	74.17 (18.06)	70.33 (21.08)	82.00 (13.08)	75.17 (16.73)
NIH-TCB (CCC), *n*		4	3	3	10
	Mean (SD)	87.56 (6.65)	83.98 (9.50)	98.00 (14.19)	89.44 (10.84)
SRS-2, *n*		6	3	2	11
	Mean (SD)	72.00 (9.65)	62.33 (4.16)	75.50 (9.19)	70.00 (9.20)
ABC-C (irritability), *n*		6	3	2	11
	Mean (SD)	10.00 (11.33)	7.33 (12.70)	2.0 (2.83)	7.82 (10.34)
Sex, *n* (*p*)		6	3	3	12
	Male Female	5 (0.83) 1 (0.17)	2 (0.67) 1 (0.33)	2 (0.67) 1 (0.33)	9 (0.75) 3 (0.25)
Race, *n* (*p*)		6	3	3	12
	American Indian	0 (0.00)	0 (0.00)	0 (0.00)	0 (0.00)
	Asian	0 (0.00)	0 (0.00)	0 (0.00)	0 (0.00)
	Black	1 (0.17)	1 (0.33)	0 (0.00)	2 (0.17)
	Hawaiian	0 (0.00)	0 (0.00)	0 (0.00)	0 (0.00)
	White	5 (0.83)	2 (0.67)	3 (1.00)	10 (0.83)
	Other	0 (0.00)	0 (0.00)	0 (0.00)	0 (0.00)
Ethnicity, *n* (*p*)		6	3	3	12
	Hispanic	0 (0.00)	0 (0.00)	0 (0.00)	0 (0.00)
	Not Hispanic	6 (1.00)	3 (1.00)	3 (1.00)	12 (1.00)

BMI: Body Mass Index; HC: Head Circumference; SB5-ABIQ: Stanford-Binet Intelligence Scales, 5th edition; NIH-TCB (CCC): National Institute of Health—Toolbox Cognitive Battery (Cognition Crystalized Composite); SRS-2: Social Responsiveness Scale—2nd Edition; ABC-C: Aberrant Behavior Checklist—Community. ^a^ From the eight subjects in Cohort 1, four agreed to be part of Cohort 2 and were re-randomized.

**Table 2:** Adverse Events (AEs) in ASD-Phen1 patients dosed with STP1 5/1 mg, STP1 10/1 mg, or placebo over a 2-week, twice a day, treatment period.

		STP1 5/1 mg (*n* = 6)			STP1 10/1 mg (*n* = 3)			Placebo (*n* = 3)		
		Mild	Moderate	Severe	Mild	Moderate	Severe	Mild	Moderate	Severe
Total Number of AEs		5	0	0	2	0	0	2	0	0
Patients with at least one AE		4 (0.67)	0 (0.00)	0 (0.00)	2 (0.67)	0 (0.00)	0 (0.00)	2 (0.67)	0 (0.00)	0 (0.00)
Gastrointrestinal disorders		2 (0.33)	0 (0.00)	0 (0.00)	0 (0.00)	0 (0.00)	0 (0.00)	2 (0.67)	0 (0.00)	0 (0.00)
	Diarrhea	1 (0.17)	0 (0.00)	0 (0.00)	0 (0.00)	0 (0.00)	0 (0.00)	0 (0.00)	0 (0.00)	0 (0.00)
	Nausea	1 (0.17)	0 (0.00)	0 (0.00)	0 (0.00)	0 (0.00)	0 (0.00)	2 (0.67)	0 (0.00)	0 (0.00)
Infections		0 (0.00)	0 (0.00)	0 (0.00)	2 (0.67)	0 (0.00)	0 (0.00)	0 (0.00)	0 (0.00)	0 (0.00)
	Infection	0 (0.00)	0 (0.00)	0 (0.00)	1 (0.33)	0 (0.00)	0 (0.00)	0 (0.00)	0 (0.00)	0 (0.00)
	Upper respiratory tract infection	0 (0.00)	0 (0.00)	0 (0.00)	1(0.33)	0 (0.00)	0 (0.00)	0 (0.00)	0 (0.00)	0 (0.00)
Psychiatric disorders		1 (0.17)	0 (0.00)	0 (0.00)	0 (0.00)	0 (0.00)	0 (0.00)	0 (0.00)	0 (0.00)	0 (0.00)
	Trichotillomania	1 (0.17)	0 (0.00)	0 (0.00)	0 (0.00)	0 (0.00)	0 (0.00)	0 (0.00)	0 (0.00)	0 (0.00)
Renal and urinary disorders		1 (0.17)	0 (0.00)	0 (0.00)	0 (0.00)	0 (0.00)	0 (0.00)	0 (0.00)	0 (0.00)	0 (0.00)
	Pollakiuria	1 (0.17)	0 (0.00)	0 (0.00)	0 (0.00)	0 (0.00)	0 (0.00)	0 (0.00)	0 (0.00)	0 (0.00)
Respiratory, thoracic, and mediastinal disorders		1 (0.17)	0 (0.00)	0 (0.00)	0 (0.00)	0 (0.00)	0 (0.00)	0 (0.00)	0 (0.00)	0 (0.00)
	Dyspnoea	1 (0.17)	0 (0.00)	0 (0.00)	0 (0.00)	0 (0.00)	0 (0.00)	0 (0.00)	0 (0.00)	0 (0.00)

**Table 3:** Pharmacokinetics characteristics of ibudilast and bumetanide in ASD-Phen1 dosed with STP1 5/1 mg and STP1 10/1 mg, twice a day, during a 2-week treatment period. Data are expressed as mean ± SD.

	Ibudilast 5 mg (STP1 5/1 mg)	*n*	Ibudilast 10 mg (STP1 10/1 mg)	*n*	Bumetanide 1 mg (STP1 5/1 mg)	*n*	Bumetanide 1 mg (STP1 10/1 mg)	*n*
Day 1								
C_max_, (ng/mL)	6.45 ± 5.48	6	18.23 ± 5.05	3	15.08 ± 9.31	6	11.45 ± 4.48	3
T_max_ (h)	4.68 ± 1.62	6	4.00 ± 1.96	3	1.76 ± 0.62	6	2.07 ± 0.09	3
AUC_0-tau_(6 h) (hrs×ng/mL)	27.21 ± 18.95	4	72.48	1	42.21 ± 22.38	5	27.21	1
AUC_0-last_ (hrs×ng/mL)	21.55 ± 17.88	6	66.09 ± 28.63	3	48.35 ± 25.00	6	37.85 ± 19.80	3
Vz/F (mL)	-	0	-	0	95,235.99	1	-	0
Cl/F (mL/h)	-	0	-	0	32,873.83	1	-	0
Day 7								
Steady-state concentration	7.94 ± 3.93	6	18.07 ± 7.08	3	na	na	na	na
Day 14								
Steady-state concentration	8.61 ± 4.33	6	19.84 ± 11.46	3	na	na	na	na
C_max_ (ng/mL)	14.95 ± 6.66	6	33.37 ± 15.52	3	21.74	6	11.09 ± 7.84	3
T_max_ (h)	3.66 ± 1.99	6	3.99 ± 2.00	3	1.54	6	1.64 ± 0.59	3
Ctrough (ng/mL)	6.16 ± 3.99	6	15.64 ± 9.70	3	1.00 ± 0.00	6	1.00 ± 0.00	3
t_1/2_ (h)	67.28 ± 38.67	6	16.71 ± 10.36	3	1.83 ± 0.50	6	1.65	1
AUC_0-tau_(6 h) (h×ng/mL)	66.86 ± 34.96	6	158.52 ± 88.37	3	50.23 ± 25.06	5	32.61 ± 14.92	3
AUC_0-last_ (h×ng/mL)/dose	1019.53 ± 875.64	6	390.48 ± 259.84	3	55.08 ± 25.06	6	33.01 ± 14.90	3
Vz/F (mL)	597,563.58 ± 552,913.71	6	397,451.90 ± 282,049.30	3	47,158.13 ± 19,241.59	6	43,460.94	1
Cl/F (mL/h)	9123.07 ± 9799.35	6	19,955.24 ± 14,255.34	3	19,349.81 ± 10,827.27	5	18,224.58	1

## Data Availability

The trial protocol and datasets used and/or analyzed during the current study are available from the corresponding authors on reasonable request due to patient privacy constraints.

## Supplementary Materials

The following supporting information can be downloaded at: https://www.mdpi.com/article/10.3390/biomedicines12071430/s1, Figure S1: Additional plots for NIH-CTB, ABC-C, and SRS-2.
