# Supplementary figures and images for: Safety, Tolerability, and EEG-Based Target Engagement of STP1 (PDE3,4 Inhibitor and NKCC1 Antagonist) in a Randomized Clinical Trial in a Subgroup of Patients with ASD
Source: Biomedicines. 2024 Jun 27;12(7):1430. doi: 10.3390/biomedicines12071430 (PMC11274259; doi:10.3390/biomedicines12071430)

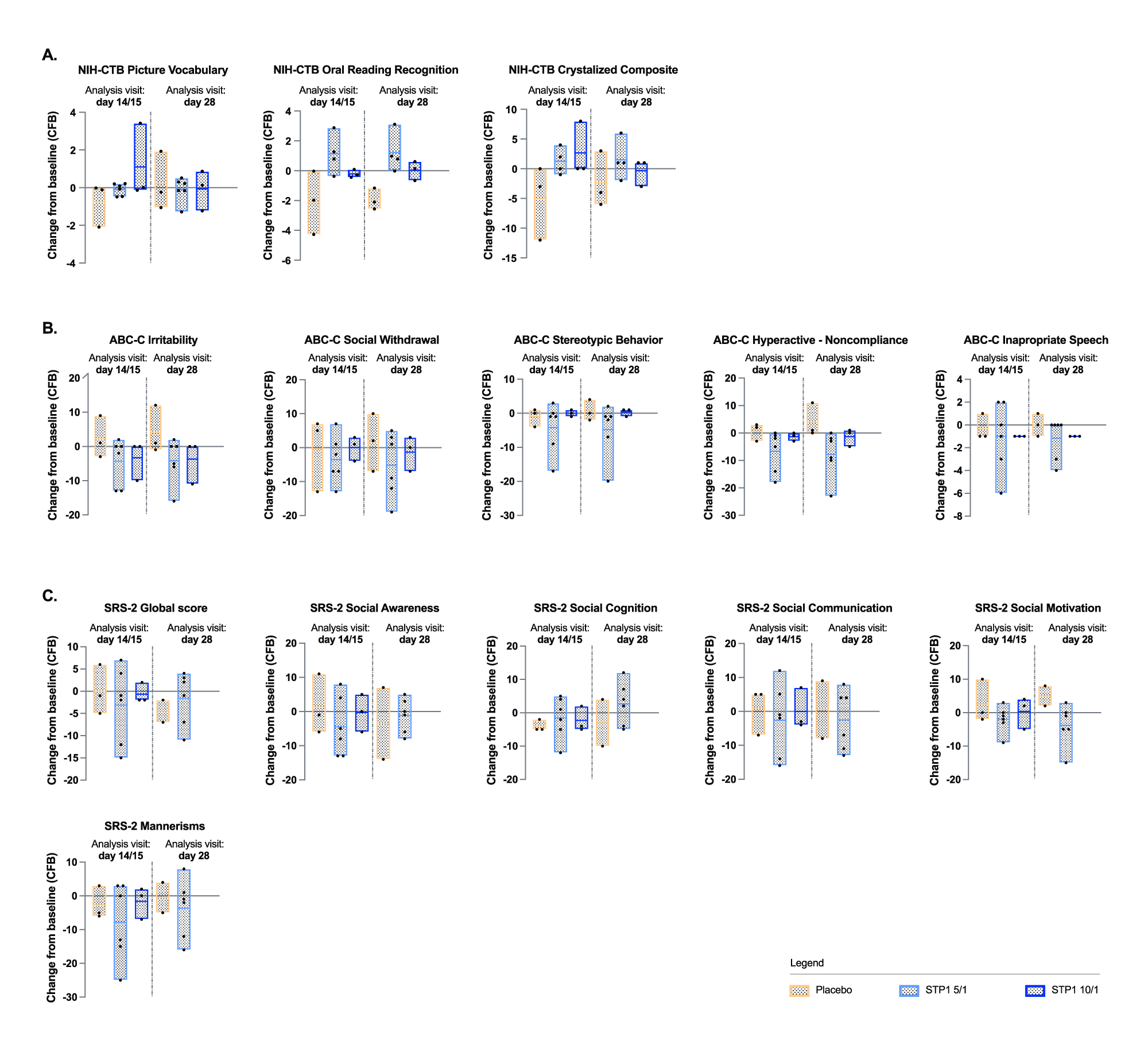

Supplement: Supplementary file 1 [file biomedicines-12-01430-s001.zip › biomedicines-2953348-Supplementalary Figure S1.jpg]
